# Author’s Responses to Peer Reviews of “Medical Brain Drain From Southeastern Europe: Using Digital Demography to Forecast Health Worker Emigration”

**DOI:** 10.2196/34077

**Published:** 2021-11-30

**Authors:** Tado Jurić

**Affiliations:** 1 Catholic University of Croatia Zagreb Croatia

**Keywords:** digital demography, Google Trends, the emigration of doctors and nurses, medical brain drain, Croatia, demography, brain drain, emigration, doctors, nurses, health care workers, health professionals, health systems, jobs, Germany, personnel, migration, workforce, medical professionals

*This is the author’s response to peer-review reports for “Medical Brain Drain From Southeastern Europe: Using Digital Demography to Forecast Health Worker Emigration.”*

## Round 1 Review

### General

The paper [1] is shortened.

I deleted the parts:

- Section Consequences of Emigration on the Ageing Population of Croatia, Serbia, and Bosnia and Herzegovina (B&H)
- Data on students for Serbia and B&H
- Tables 1 and 5
- I deleted 3000 words and added 3200 new words according to suggestions of reviewers
- Inserted data on visas for Serbia and B&H
- Modified graphs: new tests

The Abstract is structured.

References are cleaned up.

More scholarly references are cited: more pertinent/related articles published in JMIR journals and elsewhere in the past 2 to 3 years.

New references added:

- Britnell M. Human: Solving the Global Workforce Crisis in Healthcare. Oxford, England: Oxford University Press 2019:19-20.
- Mara I. Doctors on the move: mobility patterns in the EU. Wiiw Monthly Report 2019;7-8:16-22.
- Tackling brain drain requires joint efforts to improve the quality of life in all EU regions. European Committee of the Regions. URL:
  https://cor.europa.eu/en/news/Pages/tackling-brain-drain.aspx [accessed 2021-07-17]
- Recent trends in international migration of doctors, nurses and medical students. OECD iLibrary. 2019. URL: https://doi.org/10.1787/5571ef48-en [accessed 2021-04-04]
- Stonawski M, Sabourin P, Bélanger A, Loichinger E, Marois G, Ghio D, et al. Demographic Scenarios for the EU: Migration, Population and Education. Luxembourg: Publications Office of the European Union; 2019:6398.
- Knez R, Martic Biocina S, Starcevic B, Moravek D, Musovic M, Sarajlic Vukovic I, et al. Migration of Croatian physicians in the global context. Medicina Fluminensis 2020 Jun 01;56(2):88-96.
  [doi: 10.21860/medflum2020_237296]

### Reviewer A [2]

- I have now elaborated on the part related to digital traces used as an indicator of migration before.

“I have concerns that some of the Google terms used indicate wider emigration rather than health worker migration.”

I harmonized the text so that it does not talk about migrations in general but only about migrations of medical staff. In this regard, I had to make new calculations and graphs:

- Figure 5. Correlation between Google search index for query “posao u Njemačkoj + medicinska sestra” (work in Germany + nurses) in Croatian and the Organisation for Economic Co-operation and Development (OECD) statistics for emigrated nurses from Croatia to Germany (annual inflow)
- Graph 5 shows that the increase in Google search for the query “posao u Njemačkoj + medicinska sestra” (work in Germany + nurses) correlates with the increase of emigrated nurses to Germany. In the following, we show that the verification can also be performed in the opposite (ie, from Croatia in German), which again gives reliable estimates.
- Figure 6. Correlation between Google Search index for query “Arbeit in Deutschland + Arzt” (work in Germany + doctor) in German in Croatia and the OECD statistics for emigrated doctors from Croatia to Germany. This example shows that in the case of emigration of doctors, the increase in the Google search query “Arbeit in Deutschland + Arzt” (work in Germany + doctor) is in correlation with the increase of emigrated doctors to Germany.
- Figure 7. Correlation between Google Search index for query “Arbeit in Deutschland + Arzt” (work in Germany + doctor) in B&H and the OECD statistics for emigrated doctors from B&H to Germany (annual inflow).
- In the case of B&H, we calculated the annual inflow of Bosnian doctors to Germany and compared these data with the Google Trends (GT) index. As in the case of Croatia, there is a positive correlation.
- Figure 8. Correlation between Google Search index for query “Arbeit in Deutschland + Arzt” (work in Germany + doctor) in Serbia and the OECD statistics for emigrated doctors from Serbia to Germany.
- Also, in the case of Serbia, the increase in Google search for the query “posao u Nemačkoj + Doktor” (work in Germany + doctor) is correlated with the increase of emigrated doctors to Germany. There is a positive linear association between the Google index and data from official statistics (OECD).
- All tested migration-related search queries that show an indication about HWs’ (HWs) emigration planning shows a positive linear association between Google index and data from official statistics (OECD): Serbia: *R*^2^=0.3381; B&H: *R*^2^=0.2722; Croatia: *R*^2^=0.4515. The increase in Google Search is correlated with the increase in the number of emigrated HWs from Croatia, Serbia, and B&H. The decrease in Google Search is correlated with the decrease in the emigration of HW.
- Reference added: Humphries N, Connell J, Negin J, Buchan J. Tracking the leavers: towards a better understanding of doctor migration from Ireland to Australia 2008-2018. Hum Resour Health 2019 May 28;17(1):36 [doi: 10.1186/s12960-019-0365-5] [Medline: 31138211]

“It would greatly strengthen your paper if you had stronger secondary/official data on the migration of nurses/doctors from Croatia/B&H/Serbia to Germany/Austria.”

I funded the data and entered it into the text.

“Can you get data on the number of Bosnian/Serbian citizens who have obtained visas to work in Germany/Austria?”

I funded the data and entered it into the text:

Table 3. Doctors in Germany from Serbia, Croatia and B&H, OECD, 2021.

- Year: 2010, 2011, 2012, 2013, 2014, 2015, 2016, 2017, 2018, 2019
- B&H: 118, 150, 165, 202, 236, 270, 327, 397, 470, 505
- Annual inflow: 32, 15, 37, 34, 34, 57, 70, 73, 35
- Serbia: 246, 292, 381, 501, 648, 826, 1026, 1236, 1364, 1504
- Annual inflow: 46, 89, 120, 147, 178, 300, 210, 128, 140
- Croatia: 137, 158, 175, 196, 254, 295, 341, 380, 412, 428
- Annual inflow: 21, 16, 21, 63, 57, 48, 46, 42, 26

“The Introduction jumps around between source and destination countries. I would suggest that the paper discusses source countries and destination countries separately.”

I did so and better structured the whole paper.

“The pandemic as a push factor is really interesting and an important issue to raise.”

Thanks for the suggestion. I emphasized that part.

“Why use the term Western Balkan if it does not include Croatia? Better to use the countries that you’re talking about (ie, Croatia, Bosnia and Herzegovinia, and Serbia).”

This term is important because research on this topic is generally neglected. In this paper, I focus not only on these countries but also on the wider area of Southeast Europe.

“I think that the World Health Organization (WHO) Global Code on the International Recruitment of Health Personnel (2010), which is mentioned on page 11, should be more central to the paper.”

Thanks for the suggestion. I emphasized that part.

“Perhaps the paper also needs to mention the WHO 2006 list or the WHO 2020 safeguard list, which lists countries with critical health care shortages.”

Thanks for the suggestion. I emphasized that part.

“In relation to Europe, the paper should connect back to the European Observatory books on HW migration.”

Thank you. I did so.

“In the Introduction, the paper should also connect with the wider literature on brain drain/health worker migration.”

Thank you. I did so.

### Reviewer E [3]

“The paper shows a disproportion between the cited references and those in the bibliography.”

Now everything is aligned.

## Round 2 Review

### Reviewer A

1. Perhaps the title should read: Medical Brain Drain From Southeastern Europe: Using Digital Demography to Forecast Health Worker Emigration.

I agree.

2. You need to be consistent in the terms used throughout the paper (title/abstract/main text).

The new term is “Croatia and the Western Balkans (WB).”

“In displaying numbers/percentages use either decimals (60.09% or 60,09%) or commas in figures, not both.”

Ok.

3. Measuring health worker mobility is a challenge for most countries (not just Croatia, Bosnia, and Serbia), which is why registration and/or visa data from the destination country is often used as a measure of health worker emigration.

I emphasized this.

4. On page 4, you say that 65,288 nurses emigrated, and I think you’re saying that there are more Croatian/Bosnian nurses in Germany than in Croatia and Bosnia; are you? Perhaps tighten up this sentence as it’s a strong statement.

When we put these data on 65,288 emigrated HW from this region in context, we can see that this is a higher number than the total number of nurses in Croatia and B&H together. Without such intense emigration in the last 10 years, the regions of Croatia and the WB would have 50% more health workforce today. It is necessary to emphasize that this staff is crucial in the fight against a pandemic.

5. On page 7, the paragraph beginning in Austria is unclear. Rewrite these sentences.

Corrected

6. In Table 3, perhaps mark the stock data versus the flow data (ie, differentiate between the overall number of nurses in Germany [stock] vs the number of nurses entering Germany [flow]).

Corrected:

Table 3. Doctors in Germany from Serbia, Croatia, and B&H, OECD, 2021.

- Year: 2010, 2011, 2012, 2013, 2014, 2015, 2016, 2017, 2018, 2019
- Overall number of doctors from B&H in Germany: 118, 150, 165, 202, 236, 270, 327, 397, 470, 505
- Annual inflow from B&H: 32, 15, 37, 34, 34, 57, 70, 73, 35
- Overall number of doctors from Serbia in Germany: 246, 292, 381, 501, 648, 826, 1026, 1236, 1364, 1504
- Annual inflow from Serbia: 46, 89, 120, 147, 178, 300, 210, 128, 140
- Overall number of doctors from Croatia in Germany: 137, 158, 175, 196, 254, 295, 341, 380, 412, 428
- Annual inflow from Croatia: 21, 16, 21, 63, 57, 48, 46, 42, 26

7. On page 11, the section beginning what could the European Union do to address the problem, I think this should be moved out of the Introduction and into the Discussion.

I did so.

8. I think you could bring one or two issues out in the Discussion that you’ve mentioned in the text already but could make more of:

- The issue of the European Union drawing health workers from EU countries (Croatia) and nearby countries (Bosnia, Serbia) is an important issue to raise in the Discussion, as it is a clash between free movement (EU free movement) and the right to health care/need to ensure a health workforce in all regions (as per the WHO Global Code and/or the UN Sustainable Development Goals).
- I think that this method is a really interesting way of generating timely data on health worker migration. During the pandemic, the *normal* ways of data collection are simply too slow (particularly when EU countries are fast tracking health workers into the European Union). Your method is a really good way of generating timely insights into *intent to migrate* among health workers. As you mention in your paper, this should be useful for policy makers (but obviously only if they respond/react to the data). And then the next question for policy makers is how can they retain health workers during a pandemic? Increased salaries? Improved working conditions? Which links nicely to the section on what the European Union can do.

New Discussion added:

From 2010 to 2020 from the regions of Croatia and the WB emigrated 65,288 HW. Without such intense emigration in the last 10 years, the regions of Croatia and the WB would have 50% more health workforce today. It is necessary to emphasize that this staff is crucial in the fight against a pandemic.

During the pandemic, the “normal” ways of data collection are simply too slow (particularly when EU countries are fast tracking health workers into the European Union). The presented method here showed a way of generating timely insights into intent to migrate among health workers. All tested migration-related search queries that show an indication about HWs’ emigration planning shows a positive linear association between Google index and data from official statistics (OECD): Serbia: *R*^2^=0.3381; B&H: *R*^2^=0.2722; Croatia: *R*^2^=0.4515. The increase in Google search is correlated with the increase in the number of emigrated HW from Croatia, Serbia, and B&H. The decrease in Google search is correlated with the decrease in the emigration of HW.

The presented method contributes in a way that proves the feasibility of predicting further migrations from Croatia, Serbia, and B&H in this specific case of HWs to Germany and Austria, which allows reliable forecasts for the future. This procedure also presents a new methodological approach to how data obtained through GT can be standardized for comparison with official databases.

The insights are particularly relevant for national and EU policy makers and can help design appropriate strategies to retain HWs. The method can enable state agencies and the government to prepare and better respond to the shortage of HW in the future and protect the functioning of the health system. Regarding the WHO report about countries with critical health workforce shortages, this paper highlights that these issues are also relevant in European countries and that the list should be updated to include the countries B&H, Serbia, and Croatia. In addition, it is emphasized that the concept of sustainability of health care systems in the European Union is unsustainable if high-income countries do not train and retain sufficient health workers to meet the need.

While this mobility is beneficial for receiving states and contributes to a well-functioning monetary union, it negatively affects the sending countries, resulting in a *brain drain* and an erosion of public finances [4]. The issue of the European Union drawing HW from the EU periphery (Croatia) and nearby countries (B&H, Serbia) clearly shows a clash between the EU free movement and the right to health care and a need to ensure a health workforce in all European regions (as per the WHO Global Code and the UN Sustainable Development Goals).

This method could be useful for policy makers, but only if they respond and react to the data. Important question for policy makers is how they can retain health workers during a pandemic. Increased salaries and improved working conditions is certainly a good way. What precisely could the European Union do to address this problem? One approach would be to strengthen fiscal transfers to the member states and countries of the European periphery that are most affected by the harmful effects of freedom of movement [5]. However, fiscal transfers can never fully compensate for the loss of population. For example, financial compensation cannot fully compensate the departure of a nurse who left a Croatian hospital and now works in Germany—until a Croatian hospital finds a replacement. Otherwise, the specific hospital will still lack a nurse, which is reflected in Croatia’s general quality of health care. That is why we proposed a compensation solution so that Germany funds centers of excellence for the education of nurses in Croatia and the WB, provided that they remain to work in their homeland for 5 years after completing their education. In this context, it is undoubtedly to welcome the appeal of the WHO that calls on high-income countries to strive for self-sufficiency through educating, retaining, and sustaining enough doctors and nurses to staff their health care systems [6].

In a situation where there is only freedom of movement of workers but not a common pension and health care system in the European Union, or a guaranteed minimum wage, nothing significant will change at the EU level. This means that the EU framework remains a structure in which the wealthy members will continue to become richer and the poor members increasingly poorer, which also applies to the whole European periphery. Moreover, with the onset of the pandemic, the situation worsened.

Without systemic regulation of this issue at the EU level, such trends of the emigration of HWs will threaten the national health system’s capacity to respond to the needs of an ageing population and possible new waves of the pandemic.

## Abbreviations

B&H: Bosnia and Herzegovina
GT: Google Trends
HW: health care worker
OECD: Organisation for Economic Co-operation and Development
WB: Western Balkans
WHO: World Health Organization
